# Detecting fatigue of sport horses with biomechanical gait features using inertial sensors

**DOI:** 10.1371/journal.pone.0284554

**Published:** 2023-04-14

**Authors:** Hamed Darbandi, Carolien Munsters, Jeanne Parmentier, Paul Havinga

**Affiliations:** 1 Department of Computer Science, Pervasive Systems Group, University of Twente, Enschede, The Netherlands; 2 Department of Clinical Sciences, Faculty of Veterinary Medicine, Utrecht University, Utrecht, The Netherlands; 3 Equine Integration, Hoogeloon, The Netherlands; University of Rome, ITALY

## Abstract

Detection of fatigue helps prevent injuries and optimize the performance of horses. Previous studies tried to determine fatigue using physiological parameters. However, measuring the physiological parameters, e.g., plasma lactate, is invasive and can be affected by different factors. In addition, the measurement cannot be done automatically and requires a veterinarian for sample collection. This study investigated the possibility of detecting fatigue non-invasively using a minimum number of body-mounted inertial sensors. Using the inertial sensors, sixty sport horses were measured during walk and trot before and after high and low-intensity exercises. Then, biomechanical features were extracted from the output signals. A number of features were assigned as important fatigue indicators using neighborhood component analysis. Based on the fatigue indicators, machine learning models were developed for classifying strides to non-fatigue and fatigue. As an outcome, this study confirmed that biomechanical features can indicate fatigue in horses, such as stance duration, swing duration, and limb range of motion. The fatigue classification model resulted in high accuracy during both walk and trot. In conclusion, fatigue can be detected during exercise by using the output of body-mounted inertial sensors.

## Introduction

Equestrian sports are under increasing attention of public opinion regarding equine well-being. Therefore, providing more insight and transparency into the physical and biomechanical demands of horses is essential. In this regard, fatigue can be considered one of the critical elements of horse performance and welfare. During training and competition, horses usually reach some level of fatigue. Exercising after certain levels of fatigue affects the performance in several ways, including coordination reduction, muscle power decrease, and slower reaction. Continuing the exercise with excessive or prolonged fatigue may result in overtraining and injuries [1]. By assigning fatigue as an indicator [2], the injuries and overtraining may be prevented.

In contrast to human athletes, horses cannot verbally express their fatigue state. Therefore, the fatigue level should be monitored throughout the exercise. A lack of proper quantitative determination may result in not receiving adequate training stimulus or recovery periods. Finding a balance between exercise and recovery periods is very difficult, yet it is essential for optimal health and performance. Several studies showed an unusual increase in exercise load results in an increased risk of injury, as the body has not adapted to the earlier exercise responses [3–5]. In addition, fatigue has several consequences on the performance [6–8], health and welfare of the horse [9]. In severe cases, fatigue can cause horses to collapse and result in sudden death during competitions [10]. Therefore, monitoring fatigue during exercise and competition is vital for injury prevention, performance optimization, and welfare improvement.

Fatigue and subsequent injuries might be prevented by understanding fatigue mechanisms and indicators [2]. In general, “fatigue” is a multifaceted and a multidimensional term, thus, lacks a consensus definition across different domains of human and equine studies, such as exercise physiology, cognitive psychology and medical practice [11, 12]. In many studies, fatigue indicator was considered as the moment that the horse “cannot maintain the pace on treadmill despite verbal encouragement” [13–24]. This indicator can be practical in a treadmill measurement setting accompanied by veterinarians. However, it is a qualitative indicator and not practical during on-field exercise or competition [25]. Monitoring and analyzing the exercise on-field is more challenging than measurement on a treadmill since multiple factors changes between measurements, which might be surface types, weather conditions, rider effects, and speed [25, 26]. In addition, the moment when a horse voluntarily halts the exercise differs inter-individually. Some may stop before the occurrence of fatigue, while others push themselves far over their limits [27].

### Fatigue assessment methods

One of the common methods for the assessment of fitness and fatigue is standardized exercise testing (SET). In general, SET evaluates the physiological responses to the exercise. A field SET should replicate the competition environment as much as possible. It usually consists of multilevel incremental exercise steps during which plasma lactate (LA), heart rate, and speed are measured. SETs have to be adapted to the discipline and competition level to present meaningful results. Therefore, a specific exercise is often added to the SET, consisting of skills related to the discipline. As a result, the intensity of a SET, which is determined by the heart rate and LA of the performers, can vary between disciplines [25, 28, 29].

Among physiological measurements during SET, the heart rate can be evaluated by equipping the horse with a heart rate monitor. However, measuring LA is invasive and discrete (since horses are stopped several times during an exercise for blood sample collection). In addition, physiological parameters can be influenced by horse emotion and stress level [30].

In addition to the physiological parameters, biomechanical features can indicate fatigue changes. As an example, stride duration increases and speed decreases due to fatigue [13, 31–34]. However, only a few biomechanical features were investigated in fatigue studies despite many features studied in performance-related literature. For instance, stride length, stride duration, and limbs angular range of motion were studied as the indicators of performance [35, 36].

By using inertial measurement units (IMU), more biomechanical features can be monitored, especially in real-time applications. IMUs have been designed for continuous measurement, in contrast to the discrete measurement of LA. They are small, non-invasive, and easily mountable on the body. By analyzing its output signals, i.e., acceleration and angular velocity, biomechanical features, specific to the point of attachment on the body, can be calculated [37]. Therefore, IMUs can be used during exercise, with the combination of scientifically validated algorithms, for monitoring the biomechanical features during exercise [38, 39].

### Approach

Assessing fatigue of sport horses using biomechanical parameters can be approached in three steps. The first step is to identify the biomechanical features that are closely correlated to fatigue. The next step is to automatically detect fatigue using the identified features while minimizing the number of body-mounted IMUs to enhance the practicality of field measurements. And the final step is to compare the values of the biomechanical features between two levels of exercise intensity (determined by LA levels). The final step is essential for understanding the effect of training intensity on biomechanical parameters. This paper takes these steps to investigate equine fatigue indicators and detect fatigue based on extracted biomechanical features from a minimum number of body-mounted IMUs.

## Materials and methods

### Study design

**Fig 1:**
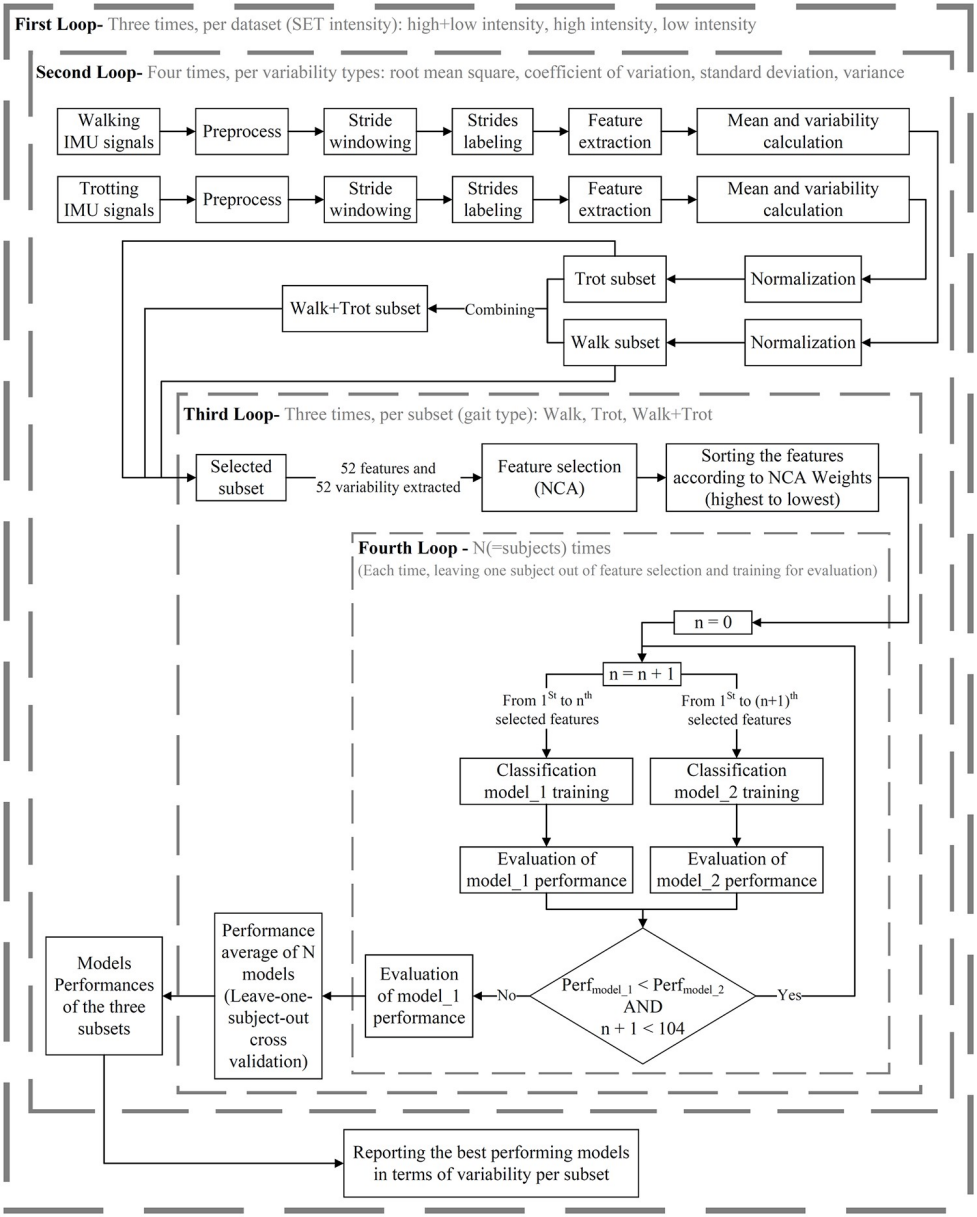
Our method for identification and evaluation of fatigue indicators.

**Fig 2:**
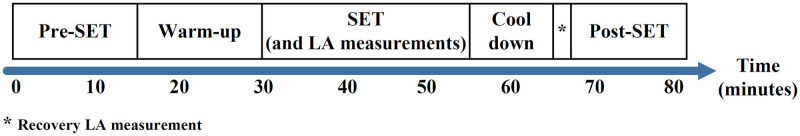
Order of the tasks for horses to perform a SET.

The proposed system for identifying and evaluating the fatigue indicators is summarized in Fig 1. In summary, all the subjects were equipped with body-mounted IMUs and performed a specific SET adjusted to their discipline. Data was collected from in-hand walking and trotting before SET (fully rested) and after SET (some level of fatigue, which were referred in this paper as pre-SET and post-SET, respectively. The sequence of tasks during SET is demonstrated in Fig 2. Subsequently, the biomechanical features were extracted from IMU signals. In this study, the fatigue state of horses during pre-SET and post-SET measurements were assigned as “non-fatigue” and “fatigue”, respectively. A Neighborhood Component Analysis (NCA) was applied to the extracted features to identify the important fatigue indicators. Finally, to quantify the importance of the selected features, they were implemented in classification algorithms, and the performances of the trained classification models were compared and analyzed.

### Study subjects

The study subjects were sixty sport horses, consisting of sixteen young Friesian stallions, twenty-eight international eventing horses, ten elite showjumping, and six elite dressage horses. For more information on the age and competition level of the subjects, see S1 Text. The inclusion criteria were horses that either performed on an international competition level or were selected for the final studbook approval test. All the subjects were examined for lameness pre- and post-SET by a veterinarian. The ones that presented lameness during the examinations were excluded from this study.

All the owners of participant horses informed written consent for research purposes. Animal Ethics Committee of Utrecht University issued the ethical permissions for the measurement of young Friesian horses. The Committee concluded that ethical approval was not required for measuring the remaining horses since it did not qualify as an animal experiment under Dutch law.

### Data collection

The data were collected from horses walking and trotting in-hand (on a hard surface) during pre- and post-SET with self-preferred speed. The assigned SET protocol for each discipline was different in terms of the specific skills tests. For more information on the SET protocol of each discipline, see S2 Text.

**Fig 3:**
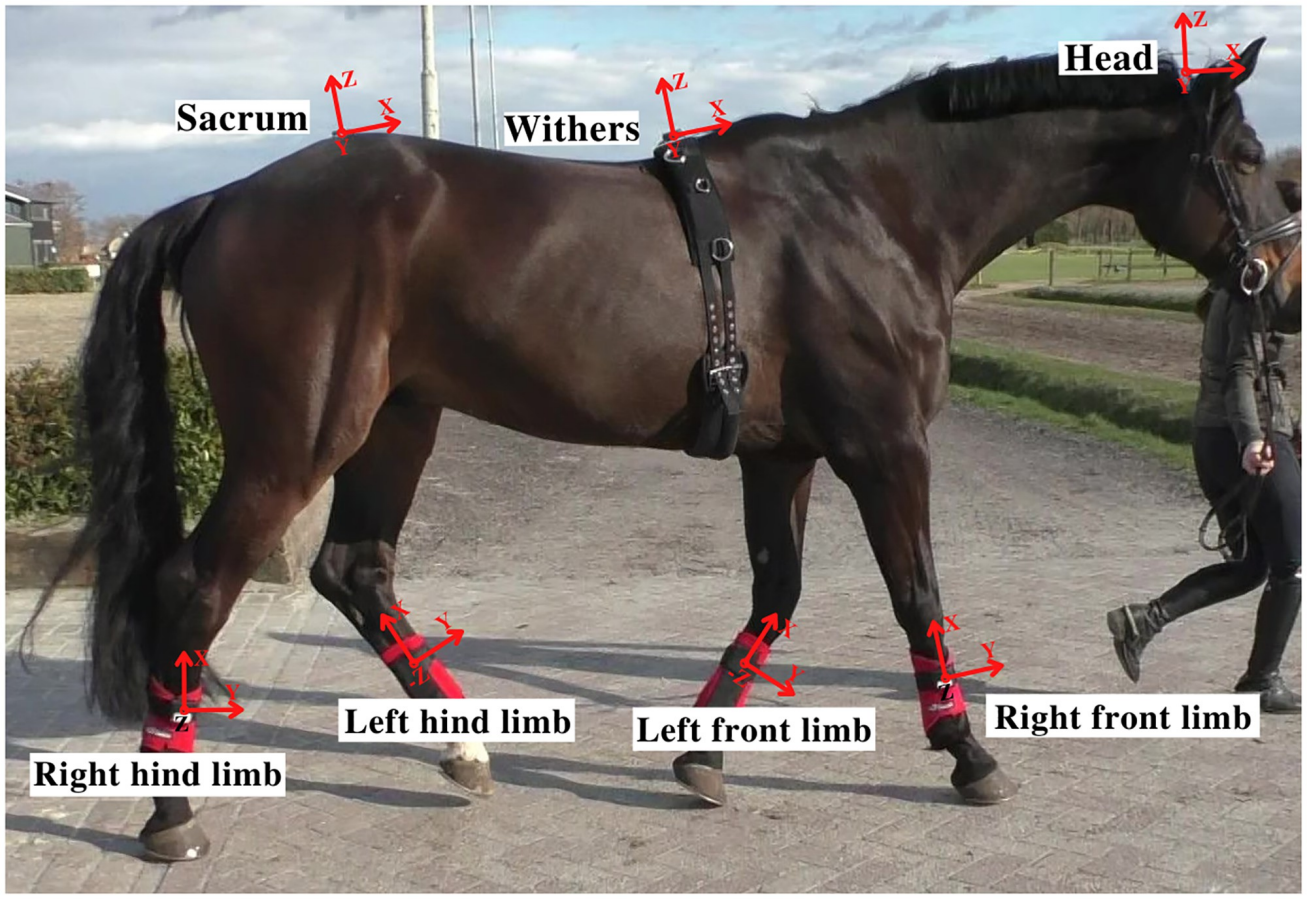
IMUs locations and orientations on horse body.

For the measurement, the horses were equipped with seven ProMove-mini IMUs [38] attached to the sacrum, withers, head (poll), and the lateral aspect of all four limbs (cannon bone). The IMUs contained a tri-axial accelerometer and a tri-axial gyroscope and were set to a sampling rate of 200 Hz, acceleration range of ±16 g, and angular velocity of ±2000 deg/s. Fig 3 demonstrates the IMU locations and orientations on the horse body. In addition to the biomechanics measurements, LA was also measured. Blood samples were taken from the jugular vein once before pre-SET, three to four times during the SET, and once before post-SET (after cool down, as recovery LA in Fig 2). After each collection, the blood sample was inserted into a portable hand-held measurement device (Lactate Pro2, Arkray Inc., Kyoto, Japan) for an instant plasma LA computation.

As demonstrated in Fig 3, the three axes of rotation for the sacrum, withers, and poll IMUs were x,y, and z, defined in the order as roll, pitch, and yaw angles. For the limbs, x, y, and z-axis were internal/external rotation, abduction/adduction, and retraction/protraction, respectively [38]. Furthermore, the three axes of horse locomotion, in general, were the longitudinal axis (aligned to the forward locomotion and parallel to the ground), the vertical axis (perpendicular to the ground or parallel to the gravitational force vector), and the mediolateral axis (perpendicular to longitudinal and vertical axes).

### Datasets and subsets

Based on the maximum LA values during SET, the relative intensity level of SET can be determined. The cut-off value for maximum LA was set at 4.0 mmol/L. This value is generally considered as the cut-off value for plasma lactate concentration in the anaerobic threshold [25]. Below and above the anaerobic threshold, we considered a low and high intensity, respectively. SET intensity was lower for show jumping and dressage horses than for young Freisian and eventing horses. Therefore, we created three datasets, which were:

- Dataset 1: Horses performed in high and low intensity SETs, which were Eventing, young Friesian, showjumping, and dressage horses (all horses in this study)
- Dataset 2: Horses performed in high intensity SET only, which were eventing and young Friesian horses
- Dataset 3: Horses performed in low intensity SET only, which were showjumping and dressage horses

The LA of all subjects pre-SET was considered low and can be indicated as normal resting values (between 0.6 and 0.8 mmol/L) [40]. Dataset 1 consisted of all horses, while datasets 2 and 3 were based on the disciplines or breeds that performed in higher and lower SET intensity levels. The SET intensity levels were defined using the maximum LA values (in Table 1), where the average for dataset 2 was more than 4.0 mmol/L, while the average for dataset 3 was less than 1.7 mmol/L. We considered the average and deviation of the datasets as the SET intensity indicators, therefore, we defined the intensity of dataset 2 and dataset 3 as high and low, respectively.

**Table 1:** Number, age (in years), and plasma lactate concentration (post-SET and the maximum value during SET) of horses by datasets.

Dataset	Number	Age	Plasma lactate concentration (mmol/l)			
		Mean (SD)	Maximum		Recovery	
			Mean (SD)	Range	Mean (SD)	Range
Dataset 1	60	9.6 (4.5)	3.49 (1.90)	0.90—10.00	1.23 (0.55)	0.60—4.00
Dataset 2	44	8.3 (4.5)	4.04 (1.80)	1.60—10.00	1.29 (0.57)	0.70—4.00
Dataset 3	16	13.1 (1.83)	1.62 (0.57)	0.90—2.90	0.98 (0.40)	0.60—2.40

As shown in Fig 1, three subsets were created from each dataset: data during walk, trot, and Walk+Trot (combination of walk and trot). By defining gaits as subsets, fatigue indicators present during each gait and independent of gait type (Walk+Trot subset) can be derived. The following steps (feature extraction, feature normalization, feature selection, and model development and evaluation) were taken on all nine subsets separately.

### Data preprocessing

The raw signals derived from the IMUs (three signals of acceleration and three signals of angular velocity) were low-pass filtered (fourth-order Butterworth filter and 30 Hz cut-off frequency) for noise reduction [38]. Then, the filtered signals were windowed into strides (from hoof-on to next hoof-on of right front limb) by implementing an estimation method on the right front limb IMU signals [39]. The pre- and post-SET data were separated from the start, hence, the strides were automatically labeled as pre-SET or post-SET.

### Feature extraction

As shown in Table 2, fifty-two features were calculated per stride, which were:

- **Gait events durations** (stride, stance, and swing duration) were determined using the hoof-on/off timings estimated from a deep learning model in a study [39].
- **Speed** was estimated using a speed estimation model from a study, which receives acceleration and angular velocity signals from the sacrum and limb IMUs and accurately estimates the speed [37].
- **Angular range of motion (ROM) of the limbs** around their three axes (protraction/ retraction, adduction/abduction, and internal/external rotation) were calculated by considering the limb as cannon bone, carpal joint as the reference point, and axes as demonstrated in Fig 3. Angular ROM of the limb were calculated using the method developed by Bosch et al. [38], where they used Valenti et. al [41] attitude and heading reference system algorithm for orientation of IMU during measurement.
- **Angular ROM of the head, pelvis, and withers** around three axes (roll, pitch, and yaw) were determined by considering the reference point as the center of the IMUs and axes as depicted in Fig 3. Angular ROM of the head, pelvis, and withers were calculated by integrating the angular velocity signals per stride and then, calculating the range (maximum minus minimum) of the integration results per stride.
- **MaxDiff, MinDiff, and displacement ROMs**: To calculate the displacement features, we applied a cyclical integration process on acceleration signals, described in [42]. MaxDiff and MinDiff are essential indicators of movement (a)symmetry and were calculated using the difference between the two peaks (MaxDiff) and troughs (MinDiff) of sacrum, withers, and head vertical (z-axis) displacement within a stride [43]. Also, the longitudinal, mediolateral, and vertical displacement ROM of sacrum, withers, head, and limbs within each stride were determined by double integration of acceleration signals per stride and then, calculating the range (maximum minus minimum) of the integration results per stride.

**Table 2:** Extracted features from strides.

Feature name	Extracted from IMU mounted on	Number
Stride duration	Right front limb	1
Stance duration	Right front limb	1
Swing duration	Right front limb	1
Speed	Sacrum, Right front limb	1
MaxDiff	Sacrum, Withers, Head	3
MinDiff	Sacrum, Withers, Head	3
Protraction/retraction range of motion (Pro/Ret)	Limbs	4
Adduction/abduction range of motion (Add/Abd)	Limbs	4
Internal/external rotation range of motion (Int/Ext)	Limbs	4
Roll angle range of motion	Sacrum, Withers, Head	3
Pitch angle range of motion	Sacrum, Withers, Head	3
Yaw angle range of motion	Sacrum, Withers, Head	3
Longitudinal displacement (horse longitudinal axis)	Sacrum, Withers, Head, Limbs	7
Mediolateral displacement (horse mediolateral axis)	Sacrum, Withers, Head, Limbs	7
Vertical displacement (horse vertical axis)	Sacrum, Withers, Head, Limbs	7
Total		52

### Feature normalization

We combined the pre- and post-SET features per subject and then normalized them to the range of 0 to 1. Intra-individual normalization helps focus on the differences between pre- and post-SET rather than on the inter-individual variations, which can depend on many factors, including the physical and fitness level. In addition, we assigned a representative of each feature per horse per trial (pre-SET or post-SET) instead of analyzing the individual strides of all horses. This helps to focus on the pre- and post-SET variations rather than individual strides differences. Therefore, the most suitable representatives of the variations are the mean and variability of the extracted features. As a definition, variability specifies the scatteredness of data points and statistically summarizes them. By other means, it can be used as a metric to check if a horse’s strides are consistent during pre- or post-SET [44, 45].

Each feature mean and variability were calculated per horse per trial (pre- or post-SET), which resulted in 104 features for each horse per trial. To find the best metrics for variability, four metrics were chosen based on the literature, which were root mean square, coefficient of variation, standard deviation, and variance [44–48] and tested on each subset (depicted as “Third Loop” in Fig 1). The best variability metric was selected according to the performance results of classification models per subset (the output of “Third Loop” into the “Second Loop” in Fig 1).

### Feature selection

The seven body-mounted IMUs were required to extract the 104 features for the model. However, equipping a horse with seven IMUs can be cumbersome for field measurements. The number of features can be reduced by selecting only the meaningful features, which may result in fewer IMUs for extracting the selected features. Selecting the important features (or feature selection) also has more advantages. It prevents the model from overfitting and increases the accuracy [37]. Hence, we implemented an NCA [49] on the features of each subset, where it assigns a weight to each feature. On the outcome of each subset, features were ranked relative to the assigned weight values. It should be noted that speed and gait events durations were calculated regardless of the selection/rejection by the proposed feature selection model considering their importance in the equine fatigue literature [13, 31–34, 36], and their outcome between pre- and post-SET were analyzed and compared.

### Model development and evaluation

According to the feature evaluation step in Fig 1, the importance of selected features per subset (walk, trot, or Walk+Trot) was quantified by testing the performance of classification models that trained solely on those features. The purpose of classification models was to classify the strides to non-fatigue or fatigue. We trained a classification model using the first selected feature by NCA as the feature for each subset and then evaluated the model performance. In the following steps, we added the next-ranked (from second-ranked to nth-ranked) feature selected by NCA and evaluated the model performance trained by a feature set consisting of the selected feature in each step and the higher-ranked features. The feature addition process was terminated as soon as the accuracy of the model decreased. Then, the features of the final feature set (that yielded the highest accuracy) were reported as the most significant fatigue indicators for that subset.

In terms of choosing the best performing algorithm, four machine learning algorithms were implemented as the model training and testing method in “Fourth Loop” (Fig 1). The tested algorithms were Support Vector Machine (SVM), k-Nearest Neighbor, decision tree, Naive Bayes, and logistic regression.

As shown in Fig 1, for each subset, a leave-one-subject-out cross-validation method was implemented in the training of classification models to make certain that a subject has been used at least one time as training and testing data, and to prevent biased results [50]. The performances of models were quantified by calculating the performance metrics (using true positive, false positive, true negative, and false negative) as follows:

- True positive (TP): The number of predictions if the true class is pre-SET and the prediction is pre-SET
- False positive (FP): The number of predictions if the true class is post-SET and the prediction is pre-SET
- True negative (TN): The number of predictions if the true class is post-SET and the prediction is post-SET
- False negative (FN): The number of predictions if the true class is pre-SET and the prediction is post-SET
- Accuracy = $\frac{TP+TN}{TP+FP+TN+FN}$
- Sensitivity or classification accuracy of pre-SET strides = $\frac{TP}{TP+FN}$
- Specificity or classification accuracy of post-SET strides = $\frac{TN}{TN+FP}$

The selected features and the performance results of the models per subset are compared in the next section. Matlab R2020a (MathWorks Inc., Natick, MA, USA) was used for all the computations for this study.

## Results and discussion

This paper investigated the possibility of detecting fatigue by using biomechanical parameters in machine learning algorithms. Important biomechanical indicators of fatigue as well as the effects of gait type and SET intensity level on the indicators were studied. Furthermore, the importance of selected indicators was determined by implementing machine learning techniques on the data and calculating their performance. It is the first time that the important biomechanical indicators of fatigue were identified using machine learning methods in equine literature. In addition, there was no other study on developing fatigue/non-fatigue classification models for horses. This study achieved highly accurate models trained with feature sets consisting of only three to six biomechanical features.

According to the definition of post-SET in the present study, horses were not pushed to exhaustion during SET. However, all horses showed some level of fatigue during post-SET, meaning that horses were not more in pre-SET condition (or resting condition), which can be reflective of some level of fatigue after SET.

In total, 3976 strides were extracted from the data of all horses (approximately sixty-six strides per horse). The number of strides per subset and the most significant features per subset, and classification models performances are presented in Table 3. The performance results and selected features are compared in the following sections.

**Table 3:** Features (mean and variability) with highest weight value based on different subsets, the best performing variability, the average performance results of the SVM classification models from leave-one-subject-out cross validation (reported as mean ± standard deviation), and the number of strides per subset.

Feature name	Datasets and subsets								
	Dataset 1 (High/low intensity SET)			Dataset 2 (High intensity SET)			Dataset 3 (Low intensity SET)		
	Walk	Trot	Walk+Trot	Walk	Trot	Walk+Trot	Walk	Trot	Walk+Trot
Event duration	Stance^M^	Swing^V^	-	Stance^M^	Swing^V^	-	Stance^M^	Swing^V^	-
Speed	-	-	-	-	-	-	-	-	-
MaxDiff	-	-	-	-	-	-	-	-	-
MinDiff	-	-	-	-	-	-	-	-	-
Pro/Ret ROM	HL^V,1^	FL^V^&HL^V^	-	HL^V^	-	FL^V^	HL^V^	HL^V^	FL^V^
Abd/Add ROM	FL^M,2^	-	FL^M^&HL^M^	-		FL^M^	FL^M^	HL^M^	-
Int/Ext ROM	-	-	-	-	-	-	-	-	-
Roll angle ROM	-	Sacrum^M^	-	Sacrum^M^	-	-	-	-	Sacrum^M^
Pitch angle ROM	-	-	-	-	-	-	-	-	-
Yaw angle ROM	-	-	Sacrum^M^	-	-	-	Withers^V^	Sacrum^M^	
Longitudinal disp.	FL^M^	FL^V^	FL^V^&HL^V^	FL^M^	FL^V^	FL^V^	FL^M^	FL^V^	FL^V^
Mediolateral disp.	-	-	-	-	FL^M^	-	FL^M^	-	-
Vertical disp.	-	-	-	Withers^M^	-	-	-	-	-
Performance results of the models based on the subsets (Mean ± Standard deviation):									
Variability metrics	VAR^3^	CV^4^	SD^5^	SD	SD	RMS^6^	SD	SD	SD
Accuracy	95±2%	83±1%	82±2%	95±2%	86±4%	80±2%	100±0%	88±2%	83±3%
Sensitivity	97±3%	80±1%	80±2%	95±2%	81±4%	78±1%	100±0%	89±2%	85±4%
Specificity	93±2%	87±2%	85±2%	95±2%	91±5%	81±2%	100±0%	88±2%	82±3%
Number of strides:									
Pre-SET	732	1234	1966	452	756	1208	280	478	758
Post-SET	782	1228	2010	451	755	1206	331	473	804

^M^ Mean of the feature,

^V^ Variability of the feature

^1^ Hind limbs,

^2^ Front limbs,

^3^ Variance,

^4^ Coefficient of variation,

^5^ Standard deviation,

^6^ Root mean square

It should be noted that if a front (or hind) limb feature is presented in the Table 3, it could be the feature that was extracted from the left or right front (hind) limb IMU. Pooling the front (or hind) limbs features allows us to focus on the feature rather than on the side. In addition, all quadrupedal vertebrates perform bilateral movement symmetry between front limbs and hind limbs [51] (including walk and trot), thus, the difference between left and right side limbs features were negligible.

According to Table 3, the selected indicators by the feature selection method were mostly gait events durations features, limbs longitudinal displacement, protraction/retraction ROM, and abduction/adduction ROM. Among the selected indicators, the longitudinal displacement of front limbs was presented in all subsets. For simplicity, the features from both front and hind limbs were considered features from “front limbs” and “hind limbs”, respectively. None of the upper body extracted features were selected from the poll (head). The angular ROMs of sacrum were selected in five subsets. The withers yaw angle ROM and vertical displacement ROM were selected as important fatigue indicators.

All the four variability metrics were reported at least once as the best performer in terms of accuracy. Standard deviation was chosen six times, while variance, root mean square, and coefficient of variation performed better each in one subset. The comparison between models performances per variability metric was reported in S1 Table.

The best performing algorithm was SVM; thus, the reported results in Table 3 were based on the SVM classification method. The performance of the models based on the other methods were reported in S2 Table. It can be inferred from Table 3 that the accuracy results of classifying walking strides using the selected walking features (95%—100%) were higher than classifying trot (83%—88%) and Walk+Trot (80%—83%) strides using their selected features.

### Comparison of the IMU locations for fatigue detection

All the subsets included at least three limb features, which presented the limbs as important locations for mounting the IMU on the body, independent of gait type and SET intensity level. In fact, by attaching one IMU to a front limb, 86% and 80% accuracy were achieved during high-intensity SET Trot and Walk+Trot, respectively. Moreover, adding another IMU on the hind limb increased the accuracy in another subset to 95% (dataset 1- Walk subset). The decrease in the number of IMUs resulting from feature selection facilitates the practicality of equipping IMU on the body.

Features extracted from poll IMU were not selected as an important feature by any model. The reason can be that horses become distracted when they are introduced to a new environment; thus, they look around and get familiar with the surroundings. Another reason could be different forces exerted by different handlers during in-hand walk and trot. Therefore, the IMU signals might get disturbed independent of the fatigue state, and the extracted features will no longer represent the horses normal head position during locomotion.

### Comparison of selected features (or fatigue indicators)

The longitudinal displacement of front limbs was the only feature present in all subsets, representing itself as an important fatigue indicator for both intensity levels and gaits. This feature is aligned with horse primary direction of movement. Therefore, it can be inferred that the longitudinal displacement of limbs can be correlated to the step length.

**Fig 4:**
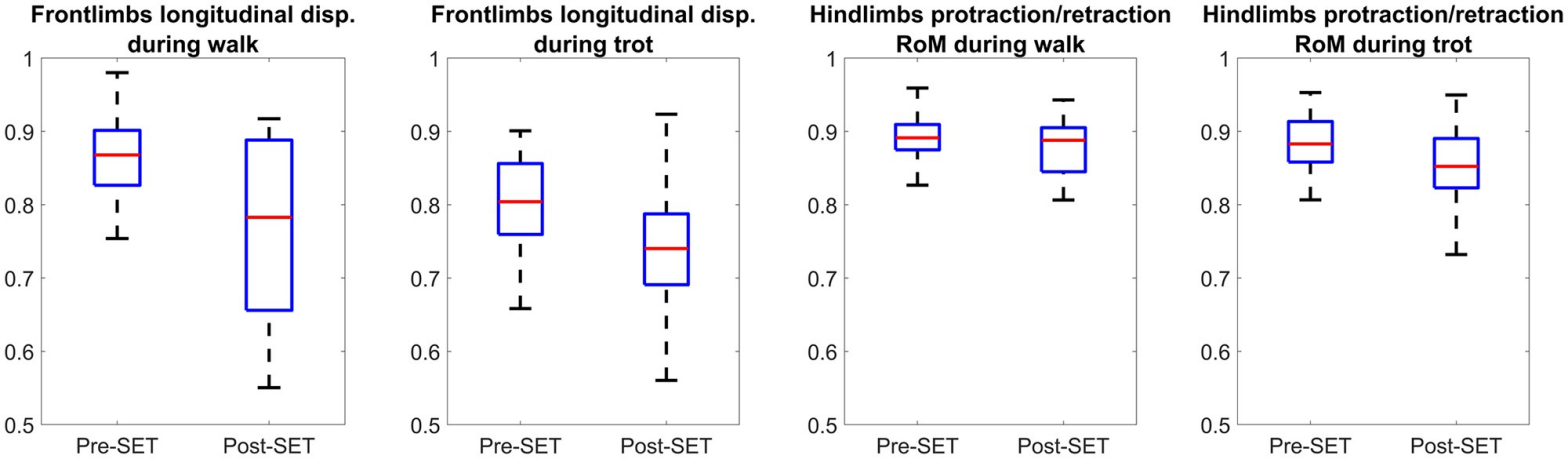
Comparison of biomechanical features of all horses between pre- and post-SET during walk and trot. The vertical axes of all plots represents the range-normalized value of the feature. The box represents the interquartile range, while the red line (horizontal line within the box) shows the median value. Each box (two for each plot) consists of one value per horse, which was averaged from all strides of the horse.

**Fig 5:**
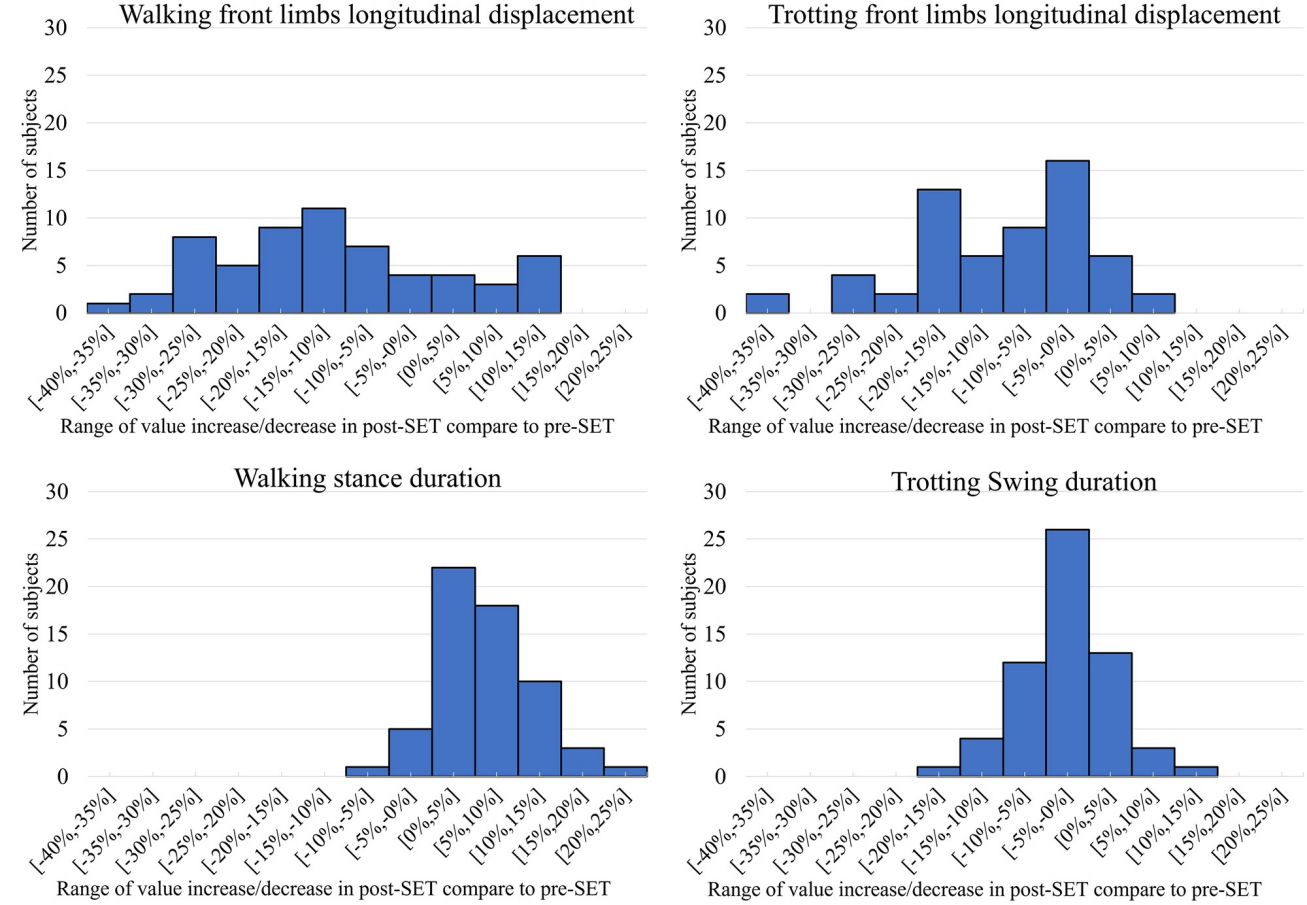
Number of horses with increased/decreased (in percentage) features values in post-SET compare to pre-SET. The vertical axes of all plots represents the range of increase (if positive) or decrease (if negative) of the specified feature value. Each bar represents the number of horses that have an increase or decrease of value within the specified range. Each plot consists of one value per horse, which was averaged from all strides of the horse.

**Fig 6:**
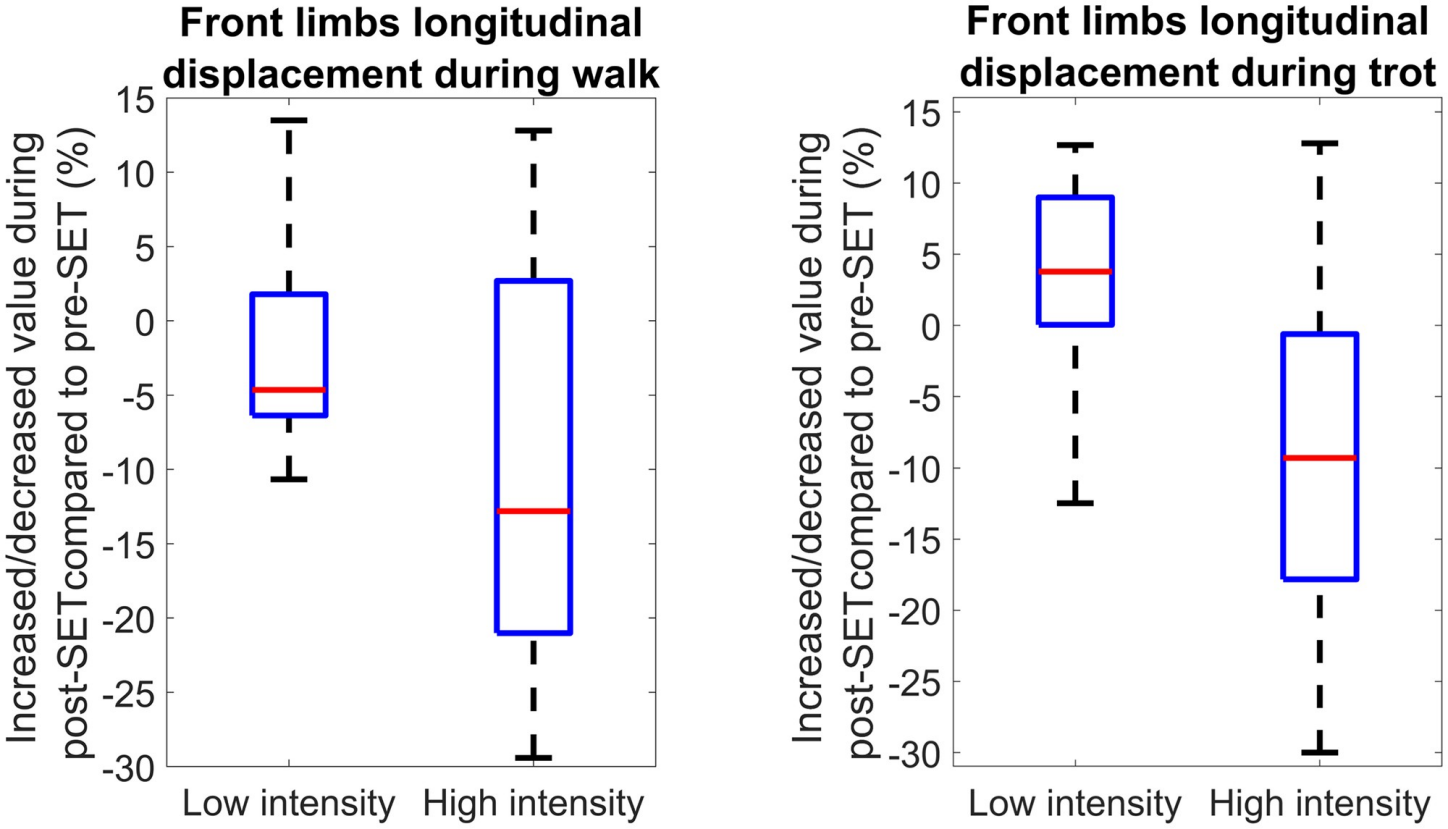
Comparison of biomechanical features between low and high intensity SETs (datasets 2 and 3) during walk and trot. The vertical axis represents the increase/decrease percentage of feature value during post-SET compared to pre-SET. The box represents the interquartile range, while the red line (horizontal line within the box) shows the median value. Each box (two for each plot) consists of one value per horse, which was averaged from all strides of the horse.

According to Figs 4 and 5, independent of SET intensity level, the front limb longitudinal displacement of forty-seven and fifty-two horses (more than 78 and 86 percent of all horses) became shorter after exercise during walk and trot, respectively. This result is aligned with the outcomes of previous studies [13, 34]. A possible explanation for the decrease of longitudinal displacement can be the decreasing of limb muscles stiffness due to fatigue [19]. Furthermore, the feature values between low and high-intensity SETs were compared in Fig 6, where the length became shorter after high-intensity SET during Walk+Trot. In contrast, the length was not shorter in all cases after low-intensity SET. It can be concluded that the horses performances might not primarily get affected by less intense exercise since the LA was low and the limb muscles were not in excessive fatigue levels.

Regardless of SET intensity and during walk or trot, the protraction/retraction angle of hind limbs appeared as an important indicator, according to Table 3. Similar to the decreasing length of limbs longitudinal displacement, the protraction/retraction angles of hind limbs were also decreased (Fig 4), which might be due to the lack of force in limb muscles caused by fatigue.

**Fig 7:**
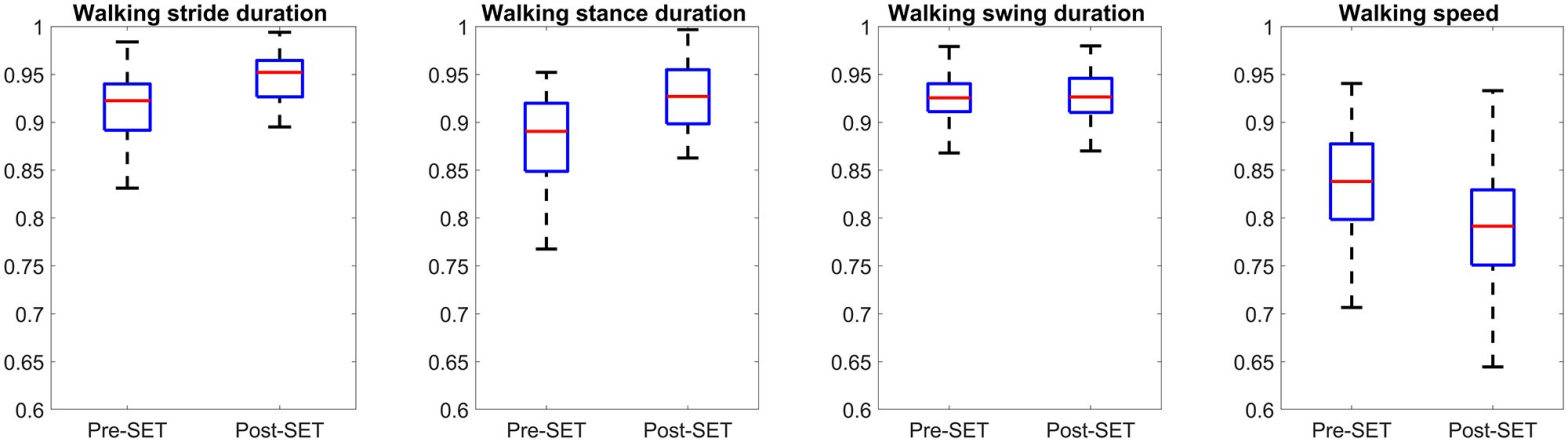
Comparison of speed and stride, stance, and swing duration features of all horses between pre- and post-SET during walk. The vertical axes of all plots represents the range-normalized value. The box represents the interquartile range, while the red line (horizontal line within the box) shows the median value. Each box (two for each plot) consists of one value per horse, which was averaged from all strides of the horse.

**Fig 8:**
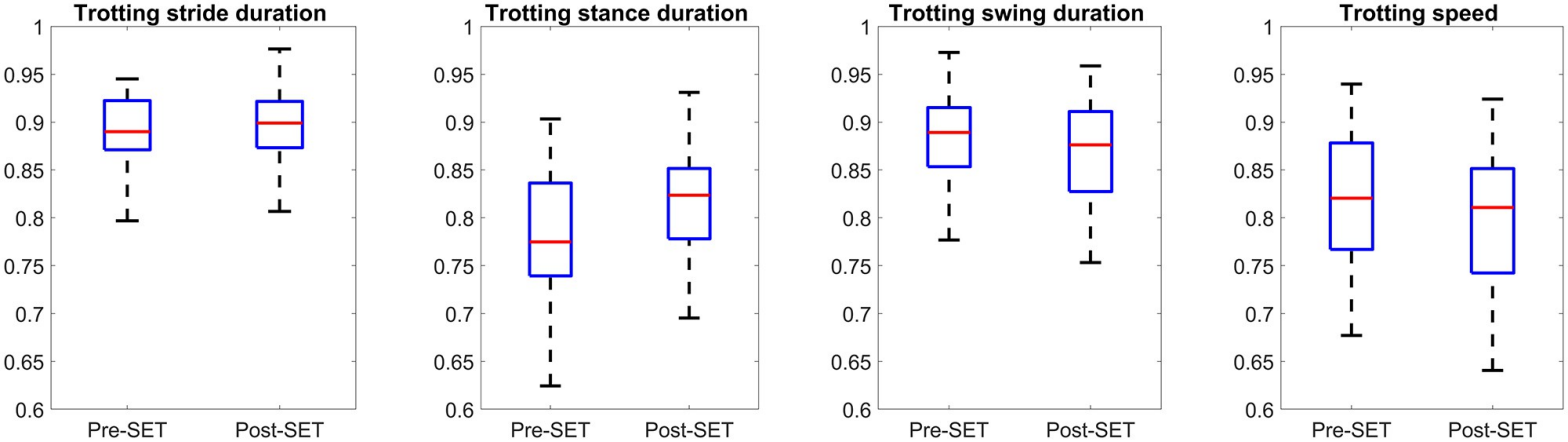
Comparison of speed and stride, stance, and swing duration features of all horses between pre- and post-SET during trot. The vertical axes of all plots represents the range-normalized value. The box represents the interquartile range, while the red line (horizontal line within the box) shows the median value. Each box (two for each plot) consists of one value per horse, which was averaged from all strides of the horse.

Stance duration and swing duration were specified as important fatigue indicators during walk and trot, respectively. Stance duration was increased in fifty-four horses (90 percent of horses) after SET during walk (Figs 5 and 7), while swing duration was decreased during the trot (Figs 5 and 8) in forty-three horses (more than 71 percent of horses).

**Fig 9:**
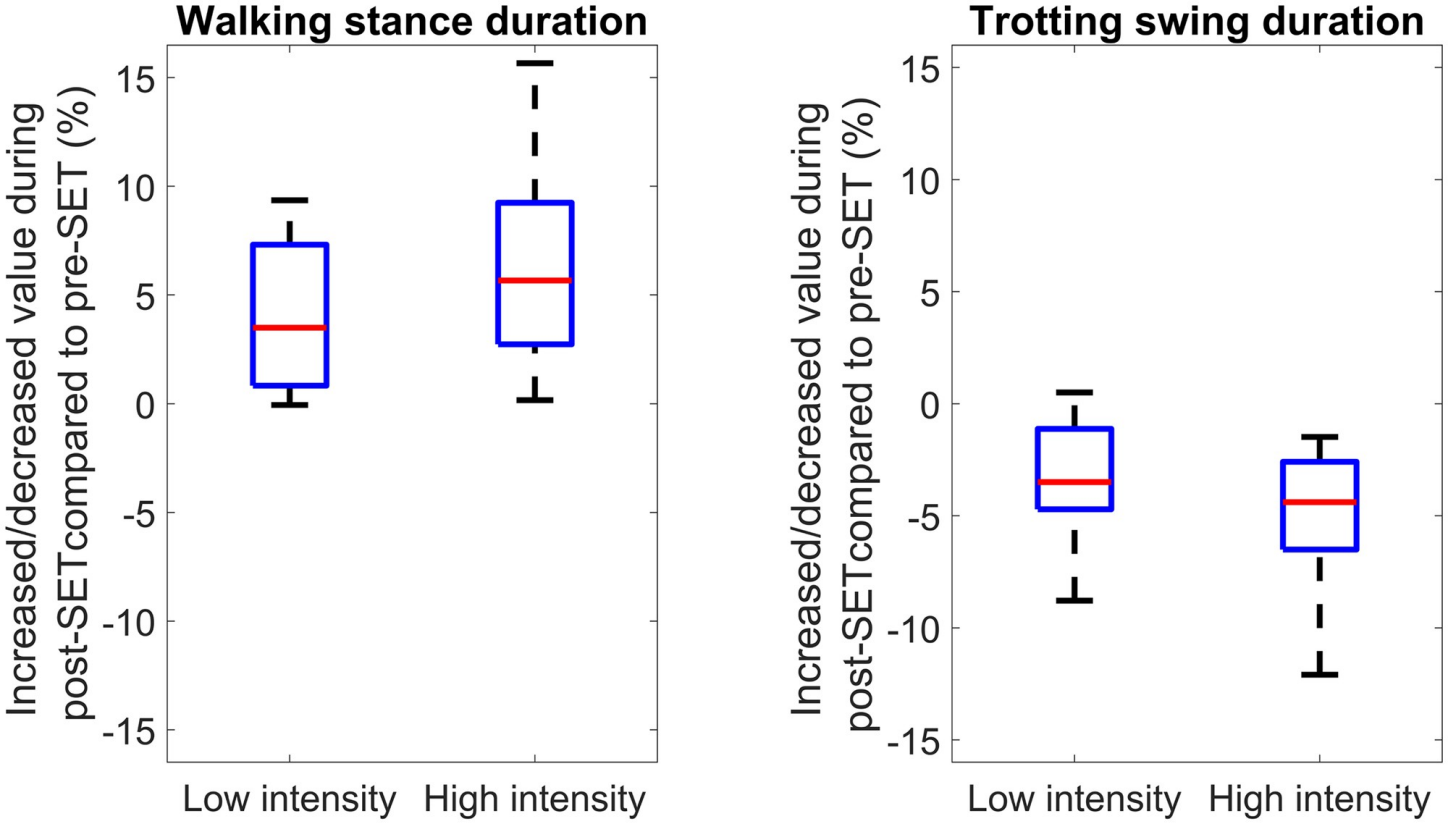
Comparison of biomechanical features between low and high intensity SETs (datasets 2 and 3) during walk and trot. The vertical axis represents the increase/decrease percentage of feature value during post-SET compared to pre-SET. The box represents the interquartile range, while the red line (horizontal line within the box) shows the median value. Each box (two for each plot) consists of one value per horse, which was averaged from all strides of the horse.

Owing to the importance of gait events features, we also investigated the other related features that the feature selection system did not select. The duration of stride, during Walk+Trot was increased, same as was reported in literature [13, 31–33, 36]. From a biomechanical point of view, the increase in stride duration is due to the decline of activity in the muscles responsible for propulsive force [34], which lets the muscle shortens with an optimal rate to output a more sustained power and more cumulative work [19, 52]. Stance duration increased in the walk as well as trot, and swing duration was approximately the same pre- and post-SET during the walk, while it was decreased during the trot. It can be seen in Fig 9 that the walking stance duration was longer, and the trotting swing duration was shorter in higher intensity SET. Combining the walking and trotting strides, stance, swing, or stride duration were not indicated as distinguishing fatigue indicators. Overall, it can be derived that the significant changes of gait events features are dependent on the gait type and independent from the SET intensity level.

### Comparison of models performances

By extracting the few selected features (Table 3) from strides, these models accurately classify horse fatigue state. The classification model trained on the walk subset of dataset 1 used no upper body features, while the models based on trot and Walk+Trot subsets of dataset 2 (high-intensity SET) used only features extracted from front limbs. The accuracy of the models were also different. For example, if both gait type and SET intensity level are unknown, the classification accuracy would be 82%. In addition, if only the SET intensity level is known, for the lower level, the model yields higher accuracy (83%) than the higher level of intensity (80%). Furthermore, if stride gait type is known, we can achieve high model performance for walk with 95% accuracy during high-intensity SET, 100% during low-intensity SET, and 95% accuracy if the intensity level is not known. In addition to walking strides, the trotting classification models based on known SET levels (86% in high intensity and 88% in low intensity) suggest better results than the model accuracy based on the mixture of high and low SET intensity levels (83%).

According to the results, the models performances in all subsets are higher for low SET intensity than high SET intensity. This can be explained due to different subjects in high and low intensity datasets. In addition, the horses disciplines are different, which can influence their gait pattern. Furthermore, the higher accuracy could be achieved if deep learning algorithms were executed. However, the low number of strides and subjects (i.e. low amount of data) could not allow for the development of deep learning models.

### Comparison to the state-of-the-art

Since there was no study on the classification of equine fatigue/non-fatigue, we compared the results with two studies on human fatigue. In one study, the walking patterns of seventeen subjects were classified as fatigue/non-fatigue induced by a squatting exercise [53]. The accuracy of the classification model was 96%, which was lower than the accuracy of low-intensity SET classification model (100%) in the current study but higher than the accuracy of the model based on all SETs. In another study, fatigue was induced by manual material handling sessions on thirty participants [54]. The result of the walking fatigue/non-fatigue classifier was 90%, which was lower than all the three walking models in this study. The mentioned studies were similar in data collection and analysis to the current study, in which they used IMU for data measurement and machine learning for data analysis. Therefore, the models reported in this paper can potentially outperform the classifiers in the human fatigue literature with a comparable study basis.

### Assumptions and limitations

It should be mentioned that for simplicity in comparing to levels of SET, we considered the SETs of eventers and young Friesian horses as “high” intensity. In exercise physiology, these SETs are submaximal SETs with moderate LA values and not defined as high-intensity levels, like maximal exercise test reaching maximal LA levels. According to the equine physiology literature, a high-intensity level SET can induce LA values as high as 32 mmol/l [55]. In general, Warmblood sport horses (including all the horses in this study) will almost never reach these high levels of LA as the nature of their disciplines is submaximal. Thus, LA higher than 4 mmol/L is considered as high intensity for Warmblood horses. There were LA value differences between SETs of different disciplines in this study, hence, we assigned the “high” intensity label to the SET of disciplines with higher LA values and “low” to those with lower LA values.

The models require at least thirty-three strides from pre/post-SET to output a valid result since they were developed using the mean and variability of the features extracted from thirty-three strides rather than single strides. For more flexibility in the classification, the development of models capable of classifying single strides should be explored in future studies.

## Conclusion

This study demonstrated that mounting only one IMU on a front limb makes it possible to monitor the value changes of important biomechanical indicators of fatigue induced by exercise. We presented walking stance duration and trotting limb longitudinal displacement as two biomechanical fatigue indicators, where most horses tend to increase and decrease respectively when fatigued. In addition, by building machine learning models on biomechanical parameters as input features, fatigue can be detected with 95% and 83% accuracy during walk and trot.

Using IMUs for sport horses apart from measuring physiological parameters during exercise can provide more objective fatigue detecting tools for riders, trainers, and officials. This may help prevent excessive fatigue and therefore, reduce injury rates. Implementing the results of this study in real-time applications can help researchers and equestrians improve the welfare of horses, enhance training sessions, and identify any level of fatigue. In future studies, the classification of horse fatigue levels using IMUs will be improved by evaluating and adding more levels of fatigue.

## Supporting information

S1 Text Subjects ages and levels of competition. (PDF)

S2 Text A short description of SET protocols used in this study. (PDF)

S1 Table Effect of different variability metrics on the performance of the models. (PDF)

S2 Table Impact of the machine learning method on the performance of the models. (PDF)
