# Supplementary material for: Detecting fatigue of sport horses with biomechanical gait features using inertial sensors
Source: PLoS One. 2023 Apr 14;18(4):e0284554. doi: 10.1371/journal.pone.0284554 (PMC10104328; doi:10.1371/journal.pone.0284554)
Supplement: S1 Text — (PDF) [file pone.0284554.s001.pdf]

S1 Text: Subjects ages and levels of competition

The average age of all the horses was 9.6 $\pm$ 4.5 years, while for Friesian horses, eventing, showjumping, and dressage horses were 3.2 $\pm$ 0.4, 11.1 $\pm$ 3.0, 12.7 $\pm$ 1.6, and 13.8 $\pm$ 1.9 years, respectively. All the horses other than the Friesian horses were competing under International Federation for Equestrian Sports (FEI) rules. The elite dressage and showjumping horses were competing at Grand Prix level and were all qualified for the Olympic games in Tokyo 2020. Moreover, six eventing horses were also qualified for these Olympic games, while other eventing horses were competing at international level (from Concours Complet International 2-star (CCI2*) to 5-star (CCI5*).
