# Supplementary material for: Detecting fatigue of sport horses with biomechanical gait features using inertial sensors
Source: PLoS One. 2023 Apr 14;18(4):e0284554. doi: 10.1371/journal.pone.0284554 (PMC10104328; doi:10.1371/journal.pone.0284554)
Supplement: S2 Text — (PDF) [file pone.0284554.s002.pdf]

S2 Text: A short description of SET protocols used in this study

In this text file, a short description of SET protocols that were used in this study is illustrated. For Friesian and eventing horses, we performed the SET protocols described in previous studies [1] and [3]. The SET protocol for the showjumping horses was adapted from two studies [4, 5]. For dressage horses, the same Friesian horses SET protocol was used with an addition of an adapted Grand Prix test [2].

References

1. Munsters CBM, van den Broek J, van Weeren R, Sloet van Oldruitenborgh-Oosterbaan MM. Young Friesian horses show familial aggregation in fitness response to a 7-week performance test. Vet J. 2013 Oct;198(1):193-9. doi: 10.1016/j.tvjl.2013.07.023.

2. Fédération Equestre Internationale (FEI). FEI dressage tests. cited 26 Jun 2021. Available from: https://inside.fei.org/fei/your-role/organisers/dressage/tests/.

3. Munsters CBM, Broek J, Welling E, van Weeren R, Sloet van Oldruitenborgh-Oosterbaan MM. A prospective study on a cohort of horses and ponies selected for participation in the European Eventing Championship: reasons for withdrawal and predictive value of fitness tests. BMC Vet Res. 2013 Sep;9:182. doi: 10.1186/1746-6148-9-182.

4. Soares OA, Ferraz G, Trigo P, D'Angelis F, Feringer Junior W, Nardi KB, Almeida F, Neto A. Comparison between specific and nonspecific tests for evaluating the physical fitness of show jumping horses. Comp Exerc Physiol. 2016 Sep;12(3):131-140. doi: 10.3920/CEP160018.

5. Munk R, Møller S, Lindner A. Effects of training with different interval exercises on horses used for show jumping. Comp Exerc Physiol. 2013 May;1(9):33-41. doi: 10.3920/CEP12016.
