# Supplementary material for: Detecting fatigue of sport horses with biomechanical gait features using inertial sensors
Source: PLoS One. 2023 Apr 14;18(4):e0284554. doi: 10.1371/journal.pone.0284554 (PMC10104328; doi:10.1371/journal.pone.0284554)
Supplement: S1 Table — (PDF) [file pone.0284554.s003.pdf]

S3 Table: Effect of different variability metrics on the performance of the models

Table 1. Effect of different variability metrics on the performance of the models (reported as mean ± standard deviation)

Datasets	Subsets	Variability Metrics			
		VAR	CV	SD	RMS
Dataset 1 (High/low intensity)	Walk	77±2%	82±2%	88±2%	95±2%
	Trot	83±1%	73±1%	77±1%	77±1%
	Walk+Trot	70±2%	72±2%	82±2%	80±2%
Dataset 2 (High intensity)	Walk	82±2%	86±2%	95±2%	88±2%
	Trot	80±4%	72±4%	86±4%	80±4%
	Walk+Trot	80±2%	72±2%	78±2%	76±2%
Dataset 3 (Low intensity)	Walk	80±2%	80±2%	100±0%	90±2%
	Trot	73±2%	73±2%	88±2%	70±2%
	Walk+Trot	75±3%	73±3%	88±3%	83±3%
