# Supplementary material for: Detecting fatigue of sport horses with biomechanical gait features using inertial sensors
Source: PLoS One. 2023 Apr 14;18(4):e0284554. doi: 10.1371/journal.pone.0284554 (PMC10104328; doi:10.1371/journal.pone.0284554)
Supplement: S2 Table — (PDF) [file pone.0284554.s004.pdf]

S4 Table: Impact of the machine learning method on the performance of the models

Table 1. Effect of different machine learning methods on the performance of the models (Mean ± Standard deviation)

Machine learning method	Datasets and subsets								
	Dataset 1 (High/low intensity SET)			Dataset 2 (High intensity SET)			Dataset 3 (Low intensity SET)		
	Walk	Trot	Walk+Trot	Walk	Trot	Walk+Trot	Walk	Trot	Walk+Trot
SVM	95±2%	83±1%	82±2%	95±2%	86±4%	80±2%	100±0%	88±2%	83±3%
k-Nearest Neighbors	72±5%	71±6%	78±4%	82±5%	70±6%	57±8%	80±3%	73±5%	73±4%
Decision tree	77±4%	73±4%	81±3%	86±2%	69±8%	71±7%	90±2%	67±9%	75±5%
Naive Bayes	82±3%	70±7%	79±4%	86±2%	78±4%	72±7%	90±2%	70±6%	68±8%
Logistic regression	88±3%	78±6%	81±2%	88±2%	78±4%	78±5%	100±0%	70±5%	73±4%
